# Advances in heterogeneity and classification of osteoarthritis

**DOI:** 10.1038/s41413-026-00549-x

**Published:** 2026-07-07

**Authors:** Taiyuan Huang, Jianxiong Shu, Zhaoran Wu, Zhong Alan Li, Rocky S. Tuan, Changhai Ding, Yao Lu

**Affiliations:** 1 Department of Joint and Orthopedics, Orthopedic Center, Clinical Research Center, Zhujiang Hospital, Southern Medical University, Guangzhou, Guangdong China; 2 Department of Biomedical Engineering, The Chinese University of Hong Kong, Hong Kong SAR, China; 3 Center for Neuromusculoskeletal Restorative Medicine, Hong Kong Science Park, New Territories Hong Kong SAR, China; 4 Institute for Tissue Engineering and Regenerative Medicine, School of Biomedical Sciences, The Chinese University of Hong Kong, New Territories Hong Kong SAR, China; 5 Menzies Institute for Medical Research, University of Tasmania, Hobart, Tasmania Australia; 6 Clinical Research Center, Beijing Tsinghua Changgung Hospital, Tsinghua Medicine, Tsinghua University, Beijing, China; 7 State Key Laboratory of Multi-organ Injury Prevention and Treatment, Southern Medical University, Guangzhou, China

**Keywords:** Bone, Diseases

## Abstract

Osteoarthritis (OA) is a highly heterogeneous disease that exhibits distinct clinical manifestations and pathological mechanisms in different joints, patients, and even at different disease stages for the same patient. Genetic and pathophysiological factors contribute to the heterogeneity of OA in clinical manifestations, treatment responses, and prognosis of patients. Despite efforts in developing disease-modifying OA drugs and treatment technologies, no current approach can efficiently delay OA progression, and results from clinical research are inconsistent due to the mismatch between treatment mechanisms and heterogeneous patient subtypes. Researchers utilize clinical data to classify OA into different phenotypes based on etiological factors, clinical symptoms, and imaging features, as well as endotypes based on biomarkers, molecular mechanisms, metabolism profiles, and omics analyses, but there is still a lack of unified standards. Therefore, a comprehensive understanding of the heterogeneity and classification of OA is crucial for stratified and personalized treatment. In this Review, we discuss the heterogeneity of OA, with an emphasis on heterogeneity in treatment responses. We provide a structured analysis of current studies of OA classification, offering new perspectives for future OA research and clinical practice.

## Introduction

As the most common chronic joint disease, osteoarthritis (OA) affects an estimated 654 million people globally and the number of OA cases worldwide increased by 132.2% from 1990 to 2020.^1^ OA typically occurs in middle-aged and elderly individuals with symptoms including pain, stiffness, swelling, and restricted joint function, resulting in a decreased quality of life, a higher risk of comorbidities, and an increased risk of mortality. In 2020, the global age-standardized years lived with disability rate for OA patients was 255.0 per 100 000, with an increase of 9.5% compared to 1990. Thus, OA places a heavy burden on the healthcare system.^2^ To date, there have been very limited treatment options for OA. The use of nonsteroidal anti-inflammatory drugs (NSAIDs) as first-line clinical management of OA-related pain is recommended by guidelines. Emerging treatment options, such as biologics, stem cell therapy, and disease-modifying OA drugs (DMOADs), have not yielded positive results in delaying OA progression.

Accumulating evidence indicates that OA is not a single, homogeneous disease, but a highly heterogeneous and complex condition involving multiple pathophysiological mechanisms and clinical manifestations. The heterogeneity of OA manifests itself not only in the diversity and differences among different patients, but also in the various stages in the same patient.^3^ This heterogeneity increases the complexity of treatment due to the mismatch between the mechanism of pharmacotherapy and the pathogenesis of specific subtypes of OA. Thus, a comprehensive understanding of the heterogeneity of OA and stratification of different patient subtypes is of great significance for developing personalized management and improving therapeutic outcomes. In this Review, we provide an overview of factors and characteristics of OA heterogeneity, summarize previous research into OA classification, and discuss the latest advances in OA research models, aiming to provide insights for future research on OA treatment and clinical practice.

## OA is a heterogeneous disease

**Fig. 1:**
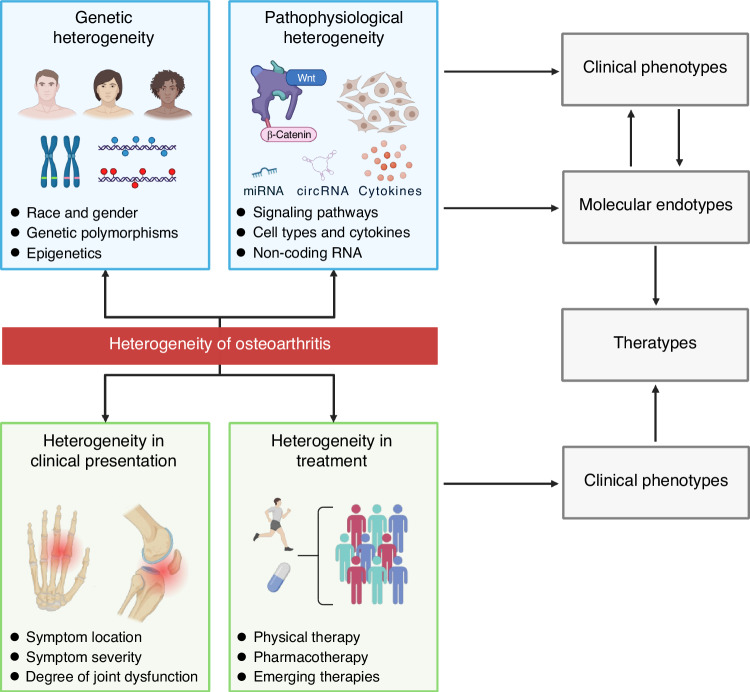
Interaction between OA heterogeneity and classification. The multifaceted heterogeneity observed in OA involves genetic, pathophysiological, and clinical aspects. These factors collectively shape the clinical phenotypes and molecular endotypes of OA patients. The heterogeneity of OA is further manifested in the variability of clinical presentations and treatment responses among different individuals, thereby influencing the observed clinical phenotypes. The clinical phenotypes and molecular endotypes jointly determine the theratypes of OA. Thus, the complexity of OA underlines the need for personalized therapeutic strategies

The heterogeneity of OA is defined as the diversity and differences exhibited by the disease in different patients, different joints, and even at different stages in the same patient. Progression of OA is influenced by a multitude of systemic and local risk factors, resulting in a significant heterogeneity among OA patients.^4^ Heterogeneity of OA is not only manifested in clinical symptoms, but also extends to the pathophysiological mechanisms, molecular levels, genetic backgrounds, environmental factors, and responses to treatment (Fig. 1).^5–9^ This highly heterogeneous feature of OA leads to diverse clinical manifestations and intricate pathogenesis.^10^ In recent years, researchers have introduced the concepts of endotype, phenotype, and therapeutic subtype (theratype) in order to better comprehend the complexity, predict the progression, and provide personalized treatment of OA.^11^ The clinical phenotype of OA, which reflects the observable characteristics of the disease, is a direct consequence of the clinical heterogeneity. It includes variations in symptoms such as pain intensity, joint stiffness, functional impairment, and radiographic changes, as well as differences in disease progression rates and responses to therapeutic interventions. The clinical phenotypes are not only influenced by structural and functional changes in the joint but are also deeply rooted in the underlying molecular and genetic mechanisms that drive OA pathogenesis. Molecular heterogeneity focuses on the specific molecular mechanisms or signaling pathways involved in the OA process. Genetic heterogeneity emphasizes the role of genetic factors in the onset and progression of OA. Other factors such as age, gender, obesity, and joint overuse can also influence disease progression, collectively contributing to the complex OA molecular endotypes. Molecular therapy aims to target the specific pathways to modulate disease progression. However, the effectiveness of such therapies is highly dependent on the molecular endotypes of OA patients. The endotype refers to class classifications based on the biological or molecular mechanisms underlying OA, particularly defined through specific biomarkers. The clinical phenotype and molecular endotype are reflected in and ultimately dictate the theratype of patients. Characterized by distinct therapeutic responses to treatment modalities, theratype is predicated on specific biomarkers, genetic profiles, or other relevant clinical parameters, enabling the identification of optimal therapeutic strategies tailored to individual patient profiles. However, achieving efficient and precise treatment for OA remains a significant challenge, necessitating a more comprehensive understanding of OA heterogeneity to enable improved patient classification and personalized therapeutic strategies.

## Genetic heterogeneity and etiological factor-based phenotypes

### Genetic heterogeneity in OA

The genetic heterogeneity of OA manifests as differences in genetic susceptibility and epigenetic regulation among different individuals or joints. This heterogeneity directly leads to diversity in disease incidence risk, clinical manifestations, and treatment responses.^12^

#### Gender-related genetic susceptibility

It has long been recognized that differences in gender-related genes play an important role in the pathogenesis of OA. The prevalence of OA among women is approximately 1.5 times that among men, with earlier onset age, higher pain scores, poorer drug response, and more significant decline in function and quality of life.^13–16^ A systematic review shows that females present more severe clinical pain, inflammation, reduction in joint cartilage volume, and physical functional impairment compared to males.^14^ Another Canadian study confirms that women have a higher prevalence of hip and knee OA, more severe symptoms, greater disability levels, and are less than one-third as likely as men to undergo joint replacement surgery, often delaying treatment until advanced disease stages.^17^

Specific genes also play different roles in male and female OA patients. For example, the T/G haplotype of the FRZB gene is associated with the pathogenesis of OA in females, but no similar findings have been found in males.^18^ Moreover, DIO2 single-nucleotide polymorphisms (SNPs) did not show an overall association with hip OA in the Rotterdam Study, but these SNPs show a trend toward reduced risk in female T allele carriers.^19^ Conversely, the COL2A1 haplotypes are associated with a decreased risk of OA in males, yet this protective effect is not observed in females.^20^

Under the influence of these genetic factors, the whole-body metabolism and joint metabolism also exhibit significant gender dimorphism.^21^ There are significant differences between males and females in terms of vitamin D metabolites, cytokines, and metalloproteinase levels in synovial fluid, as well as in the responses of chondrocytes and osteoblasts to hormones. For example, hormones regulate the expression of leptin and its receptor isoform (Ob-Rb) mRNA, resulting in significantly higher circulating leptin levels in women than in men.^22,23^ As a key hormone regulating energy balance, leptin is not only a regulator of fat metabolism but has also been shown to act as a pro-inflammatory adipokine involved in inflammation, immune regulation, and cartilage metabolism.^22^ In female patients, gender-related genotypes may indirectly regulate leptin signaling pathway activity by influencing estrogen levels, exacerbating cartilage metabolic imbalance, and inflammatory cascades.^23^ This may explain the clinical phenomenon of women being more prone to developing leptin metabolism-related phenotypes. This provides insights for the development of gender-specific preventive and therapeutic approaches.

#### Racial genetic susceptibility

Racial factors have also been found to impact the incidence, clinical presentation, and treatment choices for OA. According to Vina et al., African American populations show an increased risk of developing OA compared to Caucasian populations, and they report more severe knee pain, stiffness, and functional limitations in daily activities.^24^ It should be noted that these racial differences are not only evident at the epidemiological level but also extend to genetic regulatory mechanisms. For example, Miyamoto validated the significant association between the DVWA SNP and knee OA in a Japanese cohort and a Han Chinese case-control cohort.^25^ However, in multiple Caucasian studies, this SNP showed no association with knee OA, suggesting that this variant may have different effects in different ethnic populations.^26,27^

#### Joint-specific genetic susceptibility

Genetic heterogeneity affects the site of onset as well. A series of hub genes, including *JUN*, *ATF3*, *FOSB*, *NR4A2*, and *IL-6* play significant roles in inflammation and cartilage degradation of knee OA.^28^ Abnormal expression of these genes leads to dysfunction of chondrocytes, thereby aggravating cartilage degradation and inflammatory responses. Previous studies have revealed many joint-specific genetic drivers. For example, the chromosome 7q22 locus participates in inflammatory responses by regulating GPR22 expression, influencing chondrocyte activation and osteophyte formation. Although this locus is significantly associated with risk and progression of knee and hand OA, the strength of the association exhibits anatomical specificity.^19^ Compared to hand OA, its association effect value with knee OA progression is higher, indicating that gene expression differs between different anatomical subtypes of OA. In addition, through large-scale genome-wide association studies (GWAS), such as the combined analysis of the Rotterdam Study and the Framingham Heart Study, researchers identified *WNT9A* as a new gene locus highly associated with hand OA, particularly thumb OA.^29^ However, the *WNT9A* variant does not exhibit the same genetic association in knee and hip OA, revealing that OA may have specific genetic mechanisms in different joints. In another study, Aubourg et al. identified over 100 polymorphic DNA variants that are closely associated with OA through large-scale GWAS.^5^ Variants are primarily located in the non-coding regions of the genome and influence the expression of target genes through complex regulatory mechanisms. The distribution and effect sizes of these polymorphic DNA variants vary among individuals, making them a significant factor contributing to the genetic heterogeneity of OA.

#### Epigenetic susceptibility

In addition to genetic sequence variations, research at the epigenetic level has opened a new perspective for understanding the genetic heterogeneity of OA. Through genome-wide epigenetics, Kreitmaier et al. found the differences in DNA methylation patterns in the cartilage and synovial tissues of OA patients, which are not only associated with the severity of OA but also vary between different tissues.^30^ Differences in DNA methylation may also provide potential biomarkers for predicting the progression of OA. For example, machine learning (ML) models based on peripheral blood DNA methylation have been successfully applied to predict the progression of knee OA,^31^ suggesting the potential of epigenetics in personalized strategies of OA prediction and treatment. Further research has integrated multidimensional data such as genomics, transcriptomics, and proteomics, and thus successfully constructed molecular quantitative trait loci (molQTL) maps in the cartilage and synovial tissues of OA patients.^5,32^ These molQTL maps can not only identify potential effect genes related to OA genetic signals but also determine significant molecular differences between high-grade and low-grade cartilage. Overall, genetic heterogeneity drives gender, racial, joint site, and clinical presentation differences in OA through sequence variation, epigenetic modifications, and multi-omics interactions, providing a biological basis for subsequent clinical phenotype and molecular genotype classification.

### Phenotypes based on etiological factors

Genetic variations are thought to be significantly associated with the risk of OA. However, the functions of these genes may differ based on an individual’s genetic background, environmental influences, and many other physiological factors, resulting in distinct OA phenotypes.^33,34^ In 2009, Herrero-Beaumont proposed that OA can be classified into three major phenotypes based on etiological, clinical, and therapeutic characteristics: genetically determined, estrogen-dependent, and aging-related.^35^ It emphasizes genetic mutations, changes in estrogen levels, and the aging process in the development of OA. Later, Mobasheri and colleagues, from the perspective of disease-driven factors, proposed various OA phenotypes, including synovitis-driven inflammatory type, metabolic type, age-driven type, cartilage-driven type, trauma-induced injury-driven type, and subchondral bone-driven type.^36^ Deveza and Loeser highlight the multidimensional nature of OA phenotypes and the need for a comprehensive evaluation across multiple dimensions, including underlying disease factors, disease prognosis, and treatment response.^37^ Based on this, other researchers and we proposed a detailed Classification of Osteoarthritis for Clinical and Research (COACH) framework, which categorizes OA into several phenotypes, including load-bearing type, structural type, inflammatory type, metabolic type, systemic factor type, and mixed type.^38^ Each type has its own specific etiologies, pathogenesis, clinical manifestations, and treatment strategies. For example, load-bearing OA is mainly caused by excessive joint loading and is commonly seen in athletes and obese individuals. The treatment should focus on reducing joint load. This classification method emphasizes the independent or synergistic effects of different etiological factors in the development of OA, providing guidance for clinical diagnosis and treatment.

## Heterogeneity in clinical presentation and clinical phenotypes

### Heterogeneity in OA clinical presentation

OA exhibits significant clinical presentation heterogeneity, with differences among patients in terms of pain location, degree of joint function limitation, symptom severity, and disease progression rate.

#### Heterogeneity in different joints

The clinical presentation heterogeneity of OA is particularly evident in different joint locations. For example, patients with knee OA often experience knee pain, swelling, and limited mobility, with these symptoms being prominent when going up or down stairs, standing for long periods, or walking.^39,40^ Knee OA often accelerates wear due to bearing body weight and frequent activity. The joint space gradually narrows and cartilage wear is increasingly severe, eventually leading to complete loss of joint function.^41^ In contrast, hand OA may primarily involve the finger joints, especially the distal interphalangeal joints (DIP-OA) and proximal interphalangeal joints (PIP-OA), presenting joint stiffness, deformity, and functional limitation. Thumb carpometacarpal OA (CMC1/TS-OA), as a special subtype of hand OA, is more related to thumb dysfunction, such as weak grip and pain.^29^ The progression of hip OA may be more insidious, initially manifesting only as mild discomfort in the groin area, but as the condition worsens, pain may radiate to the thigh or buttock.^41^ Progression of OA varies among patients, with some maintaining stable conditions over time, while others may experience rapid deterioration, leading to severe joint damage and functional loss. For example, compared to non-erosive OA, patients with erosive hand OA exhibit more significant joint pain, swelling, and functional impairment, and are often accompanied by inflammatory responses and joint erosion.^42^

#### Heterogeneity in disease progression

In terms of cartilage damage in knee OA, Roemer et al. analyzed the MRI data from Kellgren-Lawrence (KL) grade 2 and 3 of knee OA patients and found that the degree of cartilage damage in patients significantly varied even within the same radiological grade.^7^ Specifically, about 21% of KL2 knees had no cartilage damage in the medial tibiofemoral joint (TFJ), while this proportion was only 5.8% in KL3 knees. But the incidence of extensive full-thickness cartilage damage was 25.3% in KL2 knees and as high as 74.1% in KL3 knees. These results indicate the variation of the prevalence, severity, and distribution of cartilage damage among OA patients.

#### Correlation between clinical presentation, pathophysiology, and imaging

The clinical presentation differences are closely linked to underlying pathophysiological changes and imaging manifestations. For instance, in knee OA, exacerbated pain often correlates with synovial inflammatory activity, objectively reflected by increased MRI-detected effusion-synovitis volume. We and others have found a significant positive correlation between effusion-synovitis changes and knee pain fluctuations.^43–46^ Specifically, when patients report aggravated pain during weight-bearing activities (e.g., stair climbing), MRI may demonstrate concurrent increases in intra-articular effusion-synovitis volume.^46^ Conversely, reduced synovitis aligns with symptom improvement. This triad of symptom variability, synovitis activity, and imaging effusion-synovitis manifestations provides an objective basis for defining clinically meaningful phenotypes. Recognizing this specific association guides clinical decisions. For such phenotypes, targeted anti-inflammatory therapies (local or systemic) may more effectively control pain than analgesics alone.^47^ Serial MRI monitoring of effusion-synovitis changes could serve as imaging biomarkers for evaluating anti-inflammatory efficacy.^48^

### Phenotypes based on clinical symptoms and imaging

The clinical presentation heterogeneity is reflected in the diverse clinical phenotypes. By leveraging the specificity of these symptoms, signs, and imaging findings, distinct clinical phenotypes can be defined.^49,50^ Classification based on clinical symptoms and imaging features represents a significant research direction.^11,51,52^ Clinical manifestations and imaging classifications are interrelated.^11,53^ Imaging techniques can reveal alterations in joint structure, which are often associated with clinical symptoms of patients.^54^ The severity of joint pain, stiffness, and dysfunction correlates with the disease severity as assessed by MRI and X-rays.^54^ Consequently, it is crucial to consider clinical features and imaging findings comprehensively when classifying OA patients.^51^

#### Phenotypes based on clinical symptoms

Statistical tools such as Latent Class Analysis (LCA) have been utilized in the field of OA clinical phenotyping. These methods employ latent variable models to categorize patients with similar symptoms and clinical presentations into distinct subtypes. For instance, Kittelson et al. used LCA to identify four pain phenotypes in knee OA: patients in Class 1 exhibited numerous comorbidities; Class 2 patients had high knee joint sensitivity; Class 3 patients experienced psychological stress and severest pain; and Class 4 patients demonstrated mild radiological manifestations, good mental health, strong muscle strength, low pain sensitivity, the youngest age, and the least functional impairment.^49^ The findings underscore the crucial roles of psychological factors, comorbidities, and joint sensitivity in the categorization of knee OA pain phenotypes. Subsequently, Vongsirinavarat further classified OA patients into four subtypes based on the degree of activity limitation: no disability, mild disability, moderate disability, and severe disability.^55^ They not only focus on the intensity of patients’ pain but also consider multiple aspects such as the range of joint motion, muscle strength, and the ability to participate in social activities, resulting in differences in clinical manifestations among subtypes. Perrot classified OA pain into three clinically meaningful dimensions: local pain, neuropathic-like pain, and deep pain by developing and validating the Osteoarthritis Symptom Inventory Scale questionnaire.^56^

#### Phenotypes based on imaging

Imaging classification has made significant progress in assessing disease severity, predicting progression, and guiding personalized treatment strategies. Traditionally, X-rays are used to evaluate joint space narrowing and osteophyte formation, which can be quantified for OA severity using the KL grading system that classifies OA from grade 0 to 4 based on the radiographic features.^57–59^ Although X-ray detection can display changes in bony structures, it is limited in finding soft tissue lesions such as cartilage and synovium, and its sensitivity to early changes.^60,61^ Magnetic resonance imaging has become an important tool to identify structural changes for the radiological classification of OA due to its high-resolution imaging capabilities of joint structures. MRI can not only clearly display bony changes such as osteophyte formation and joint space narrowing but also accurately assess soft tissue lesions such as cartilage degeneration, bone marrow edema, and synovial inflammation.^62^ Although these structural changes do not directly equate to the OA phenotype, the structural alterations identified by MRI can serve as important imaging biomarkers in OA phenotype research.^50^ Roemer et al. introduced the Rapid OsteoArthritis MRI Eligibility Score (ROAMES) system to provide a standardized assessment framework for MRI-based radiological classification of OA. The ROAMES system uses a tri-compartmental anatomic approach to meticulously score different regions of the knee joint, including cartilage, meniscus, synovium, and osteophytes. Five major OA structure-related phenotypes were identified: inflammatory phenotype, meniscus-cartilage phenotype, subchondral bone phenotype, atrophic phenotype, and hypertrophic phenotype.^50^ Recently, the longitudinal study by Liu et al. revealed a close link between MRI-based structural phenotypes and short-term structural changes as well as subsequent total knee replacement surgery.^63^ Inflammatory and subchondral phenotypes are significantly associated with the structural progression of knee OA, and patients with these phenotypes are more likely to undergo total knee replacement (TKR). In contrast, the meniscus-cartilage phenotype has a weaker association with TKR. Meanwhile, overlapping phenotypes are common in knee OA patients and may increase the risk of disease progression and TKR. Synovitis is an important component of the OA process, especially regarding the inflammatory phenotype of OA. Contrast-enhanced (CE) MRI, particularly dynamic CEMRI, is ideal for assessing synovitis. Exudative synovitis and Hoffa’s synovitis are often used as surrogate markers for synovitis in epidemiological observational studies of knee OA.^64^ It is shown that synovitis is present before the onset of radiographic OA and its signs gradually intensified as radiographic OA approached, suggesting that synovitis may be an early harbinger of OA.^65^ This may help to identify OA patients who are more likely to belong to the inflammatory phenotype.

In recent years, AI-driven patient stratification models have shown great potential in OA, providing powerful tools for understanding disease heterogeneity and enabling precise diagnosis and treatment. These models use radiomics data to construct dynamic, individualized disease trajectory prediction frameworks, which help identify patient subgroups with unique disease trajectories and pathological changes. Specifically, machine learning (ML) models have made remarkable advancements in the diagnosis of OA using MRI. Through combined high-resolution MRI with techniques such as feature selection, extraction, and texture analysis, ML can be used to diagnose and predict OA progression.^66^ Our group has revealed that the three-dimensional texture features of the infrapatellar fat pad (IPFP texture score) serve as an objective and effective imaging biomarker, capable of quantifying changes in the subchondral bone microenvironment. This provides a critical input for developing AI-driven risk stratification models for knee OA.^67^ We also integrate a neural network model (JS-RM) incorporating multi-structural MRI radiomics features of the meniscus, femoral cartilage, and tibial cartilage, achieving high-precision prediction of radiographic knee OA onset.^68^ This model significantly enhances the ability to identify high-risk patients with specific early degenerative joint structure features (improved from 0.474 to 0.874, sensitivity from 0.586 to 0.812).^68^ Bowes et al. developed a B-score quantification metric based on statistical shape modeling using ML. Compared to the KL grading system, the B-score provides greater precision and finer grading through precisely quantifying the characteristic 3D bone shape of OA, effectively predicting clinically significant outcomes such as pain, functional limitation, and total knee replacement in individual patients.^69^

AI-based models can also be used to analyze multiple data types. Castagno et al. integrated clinical, biochemical, X-ray, and MRI data to develop an automated ML tool for the prediction of rapid progression in knee OA, especially in young patients and early OA.^70^ A three-stage method proposed by Niamh Belton’s team is also representative. Based on the principle of anomaly detection, this study uses deep learning to construct a normal representation of healthy knee X-ray images, and then automatically assesses the severity of the disease by quantifying the degree of deviation between individual images and the normal center. Compared to traditional KL grading and OARSI grading systems (with inter-observer reliability ranging from 0.51 to 0.89, and ambiguous terms like “suspicious” and “possible” exacerbating assessment discrepancies), this method effectively reduces reliance on discrete quantitative categories, enabling continuous grading.^71^ Park et al. used ML techniques to conduct an in-depth analysis of OA progression patterns, combining demographic factors (age, BMI, bone density) and imaging factors (joint space narrowing, osteophyte grade) to develop a high-performance three-class prediction model. They identify patients into unicompartmental OA and tricompartmental OA subtypes. Tricompartmental OA subtype can be further identified as tricompartmental OA dominated by joint space narrowing and tricompartmental OA dominated by osteophytes. This model reveals that metabolic diseases are associated with tricompartmental OA with large osteophytes, and patients with osteoporosis may progress to tricompartmental OA dominated by joint space narrowing, while patients with high BMD may progress to single-compartment OA.^72^ Therefore, these AI-assisted models reveal tissue microheterogeneity and complex patterns that are difficult to capture using traditional methods, providing more precise radiomic biomarker support for clinical phenotypes based on structural changes in imaging. They emerge as powerful tools for patient risk stratification, prognosis prediction, and personalized treatment decisions, effectively promoting the clinical translation of big data and artificial intelligence in precision medicine for OA.

## Pathophysiological heterogeneity and molecular endotypes

### Heterogeneity in OA pathophysiology

OA variability is reflected in not only genetic susceptibility but also molecular and pathophysiological diversity. A central aspect of this heterogeneity is the inconsistent roles of signaling pathways across different joint locations and at different disease stages. Understanding of these molecular players is critical for deciphering OA pathogenesis and advancing toward precision medicine.

#### Signaling pathway

Various signaling pathways are involved in OA, including the ① wingless/integrated/beta-catenin (Wnt/β-catenin), ② nuclear factor-κB (NF-κB), ③ focal adhesion, ④ phosphatidylinositol 3-kinase/protein kinase B/mammalian target of rapamycin (PI3K/AKT/mTOR), ⑤ adenosine monophosphate-activated protein kinase (AMPK), and ⑥ growth factor transforming growth factor β (TGF-β)/bone morphogenetic proteins (BMP) pathways.^8,73,74^ Regulators such as hypoxia-inducible factors (HIFs), fibroblast growth factor (FGF), runt-related transcription factor 2 (Runx2), and interleukins (ILs) are also involved. These signaling pathways and regulators play different roles in OA of different joint locations and at different stages of OA (early, middle, and late). For example, the Wnt/β-catenin signaling pathway in temporomandibular joint OA leads to significant morphological defects and changes in cell activity through the activation of β-catenin signaling, such as reduced chondrocyte proliferation, increased apoptosis, and upregulated expression of matrix-degrading enzymes, mimicking an OA-like phenotype.^75^ In hip OA, the overexpression of β-catenin is associated with severe cartilage degeneration, subchondral sclerosis, and osteophyte formation.^76^ However, in spinal joint OA, elevated β-catenin signaling is associated with increased pain sensitivity and the transcriptional activation of OA pain-related factors, indicating its important role in spinal tissue homeostasis and degeneration.^77^ The PI3K/AKT/mTOR pathway is essential for normal metabolism of joint tissues, but is also involved in development of OA.^78,79^ Similarly, TGF-β3 can promote the proliferation and differentiation of chondrocytes to maintain cartilage homeostasis, but the content of TGF-β3 abnormally increases at the late stage of OA and leads to chondrocyte hypertrophy and apoptosis, and is positively correlated with OA symptoms such as pain.^80^

Molecular drivers of OA pathophysiological heterogeneity converge on core pathways that define actionable endotypes: The mTOR pathway exemplifies inflammation-cartilage crosstalk—beyond regulating chondrocyte autophagy and hypertrophy in cartilage-destructive endotypes, it amplifies synovial inflammation via macrophage HIF-1α/VEGF activation. Similarly, the NF-κB pathway integrates inflammatory (synovial cytokine storms) and structural damage (matrix metalloproteinase-induced by cartilage degradation), while the Wnt/β-catenin pathway exhibits joint-specific duality (e.g., mechanical osteophytogenesis in hips and neuropathic pain in spine). Prioritizing these signaling pathways can establish a unified framework that links molecular features with clinical classifiers, replacing static catalogs with hierarchical mechanisms.

#### Cytokines

The expression patterns and activities of cytokines also vary among different OA patients and joints. For example, significantly different expression levels are observed for multiple cytokines, such as EGF, FGF2, MCP3, MIP1a, and IL8, in the serum of OA patients from different joints, such as the hip and knee joints.^81^ The expression levels of pro-inflammatory cytokines in synovial fluid, such as interleukin-6 (IL-6), can vary by thousands of times among different OA patients.^82^ The infiltration of CD14^+^ macrophage and the concentrations of cytokines such as IL-4, IL-10, and TNF-α also vary in different joints.^83^

#### Synovial inflammation

Labinsky et al. revealed the heterogeneity in synovial inflammation among OA patients via multiparametric analysis.^82^ The severity of synovial inflammation, the types of cells involved, and the expression patterns of cytokines vary from patient to patient. This heterogeneity is reflected in the degree of infiltration of immune cells, such as macrophages and T cells, as well as the functions of non-immune cells, such as mesenchymal cells. Synovial fibroblasts also exhibit high diversity and functional specificity in OA. Through single-cell RNA sequencing, researchers identified seven functionally distinct fibroblast subpopulations in healthy and injured synovium, including Angptl7^+^ cells with neurogenic-related characteristics, αSMA^+^ cells with myofibroblast-like characteristics, and IL-6^+^ cells with pro-inflammatory properties.^84^ Subpopulations show different changes in proportion and number after joint injury, especially Prg4hi fibroblasts, which proliferate significantly in OA and specifically secrete R-spondin 2 (Rspo2) to activate the Wnt/β-catenin signaling pathway, thereby driving pathological changes in the synovium, such as inflammation, fibrosis, and osteophyte formation.

#### Non-coding RNAs

In addition, non-coding RNAs such as microRNAs, circRNAs, and snoRNAs are important in the pathogenesis, and their differential expression contributes to OA heterogeneity. For instance, Peffers et al. identified a set of differentially expressed snoRNAs associated with cartilage aging and OA by using microarray and qRT-PCR techniques. In vitro results show that the knockdown or overexpression of snoRNAs significantly altered the phenotype of chondrocytes.^85^

### Endotypes mediated by OA pathology

Distinct molecular heterogeneity of OA is associated with unique biological pathways and molecular processes. A thorough investigation of these mechanisms facilitates the identification of specific biomarkers or characteristic molecular alterations in OA, which are critical for the classification of molecular endotypes.^86^ At the same time, the precise categorization of OA molecular endotypes deepens the understanding of OA pathogenesis, offers novel insights and methodologies for early diagnosis and prognostic evaluation, and provides a foundation for the development of targeted therapies aimed at specific molecular mechanisms.

#### Endotypes based on biomarkers

Biomarkers are playing an increasingly important role in the diagnosis, classification, and prognostic evaluation of OA.^87–89^ Different endotypes of OA patients present unique biomarker characteristics, which cover multiple aspects such as inflammatory mediators, cartilage degradation products, bone metabolism markers, and factors related to metabolic syndrome. According to the latest research by Mobasheri et al., OA can be classified into four main molecular endotypes: inflammatory type, subchondral bone remodeling type, metabolic syndrome type, and aging-related type.^86^ In patients with inflammatory OA, the levels of inflammatory factors (e.g., IL-1β, IL-6, and TNF-α) are increased.^90^ This reflects the key role of inflammation in OA progression. Subchondral bone remodeling OA is characterized by elevated CTX-I level, indicating that increased bone resorption activity is an important pathological process in this endotype of OA. Metabolic syndrome-related OA is characterized by abnormal expression of bone metabolism markers and factors related to metabolic syndrome, revealing the role of metabolic factors in the pathogenesis of OA^86,91^. Aging-related OA shows characteristics such as decreased solubility and proteolytic digestibility of the extracellular matrix of articular cartilage, and accumulation of pentosidine, which are closely related to aging physiological processes.

The use of advanced algorithms, such as the Subtype and Stage Inference (SuStaIn) model, to conduct in-depth analysis of the biomarkers of patients improves the identification of OA endotypes. The study by Lv and colleagues classified OA into three subtypes through SuStaIn: early-stage pain type, structural lesion with pain type, and late-stage pain type. Each endotype shows a unique pattern of biomarker changes during the OA progression.^92^ In terms of biomarkers such as WOMAC pain score, joint space width (JSW), cartilage thickness, and osteophytes. Lv et al. based on the characteristic changes of representative molecules over time, classified knee OA into pre-stage, early-stage, progressive, and end-stage. They further divided advanced knee OA into cartilage degradation-driven type, bone remodeling-driven type, inflammation-driven type, and pain-driven type based on the core pathophysiological characteristic.^92^ Cartilage degradation-driven type is characterized by the imbalance of synthesis and degradation of cartilage matrix, and the increase of cartilage degradation markers reflects the loss of cartilage in this process. Bone remodeling-driven type emphasizes the role of bone formation and resorption in knee OA, and the changes in bone remodeling markers indicate the degree of bone metabolism activity. Inflammation-driven type highlights the importance of chronic, low-grade inflammation in the pathogenesis of knee OA, and the increase of inflammatory markers is closely related to the activation of the innate immune system. Pain-driven type is directly related to the patient’s pain experience, which may be associated with bone marrow lesions and synovitis. This classification may provide guidance for preventive and intervention measures at different stages of OA. For example, in the pre-stage, detecting molecules related to risk factors (e.g., adipokines) may help to identify high-risk populations early.^93,94^ In the early stage, detecting molecular markers such as microRNAs (miRNAs) and IL-17 could provide evidence for diagnosis. In late-stage knee OA, specific molecular markers (e.g., NLR and let-7e) show potential value in predicting disease progression and assessing treatment effects.^95^

The introduction of ML technology also revolutionizes the analysis of biochemical markers. Angelini et al. recently developed a method based on unsupervised machine learning (specifically clustering) to identify three main endotypes of OA patients in the Innovative Medicines Initiative (IMI)-APPROACH patient cohort for knee OA, using a set of 16 biochemical markers associated with different joint tissue processes (e.g., degradation, formation, or inflammation): low tissue metabolism type (C1), structural damage type (C2), and systemic inflammation type (C3).^96^ Each subtype has unique physiological characteristics. For example, patients with low tissue metabolism exhibit lower levels of biochemical markers associated with inflammation and structural damage, while patients with structural damage exhibit higher levels of bone formation/resorption and cartilage degradation markers. The systemic inflammation subtype exhibits characteristics of inflammatory responses and joint tissue degeneration. Through this machine learning method, they found that C1 has the highest proportion of non-progressors, structural damage patients are more likely to be associated with longitudinal structural progression, and systemic inflammation patients are more likely to experience persistent or progressive pain.^96^ Additionally, a large-scale study by Nielsen et al. integrated extensive multimodal data from the UK Biobank and utilized interpretable ML models to predict the five-year diagnosis risk of OA through identifying a series of critical OA risk biomarkers.^97^ This study included approximately 20 000 OA patients and 20 000 health controls, and revealed that age, body mass index, and NSAIDs prescription were the primary factors in OA risk prediction. Moreover, this study identified 14 OA subgroups with distinct risk biomarker profiles and formulated simple clinical association rules for these subgroups.

#### Endotypes based on molecular mechanisms

The development of OA involves multiple pathophysiological mechanisms and signaling pathways involving skeletal remodeling, cartilage erosion, pain, synovitis, low-grade inflammation, and metabolic syndrome.^98^ Dell’Isola performed a systematic literature review of 25 studies and identified six major OA endotypes.^99^ The chronic pain endotype is characterized by central sensitization mechanisms dominating the pathophysiology of the disease. The inflammatory endotype shows high levels of inflammatory biomarkers. The metabolic syndrome endotype is closely related to metabolic disorders such as obesity and diabetes. The bone and cartilage metabolism abnormality endotype involves changes in local bone and cartilage metabolism. The mechanical overload endotype is mainly caused by joint mechanical abnormalities, such as joint deformities and medial compartment lesions. Finally, the minimal joint disease endotype is characterized by mild clinical symptoms and slow disease progression.^99,100^

The interaction between inflammation and cellular senescence in OA has attracted growing attention. The pathogenesis of OA is accompanied by the continuous presence of intra-articular inflammation and the acceleration of chondrocyte senescence. Han et al. classified OA into inflammation-dominant and cellular senescence-dominant endotypes.^39^ The inflammation-dominant OA endotype presents high levels of inflammatory cytokines and synovitis within the joint. These factors promote the degradation of cartilage matrix and joint damage.^101^ The cellular senescence-dominant endotype focuses on the role of chondrocyte senescence in OA progression. Cellular senescence leads to decreased chondrocyte function and senescence-associated secretory phenotype (SASP), exacerbating the inflammatory response and matrix destruction.

With the rapid advancement of high-throughput sequencing technologies and bioinformatics methods,^102^ transcriptomic classification has become an essential tool for revealing the heterogeneity of OA.^103^ Through transcriptomic sequencing of knee cartilage, subchondral bone, and synovial tissues, Yuan et al. classified OA patients into four subtypes: the glycosaminoglycan metabolism disorder subtype (C1), the collagen metabolism disorder subtype (C2), the activated sensory neuron subtype (C3), and the inflammatory subtype (C4).^104^ At the molecular level, these subtypes exhibit significant differences. For example, the C1 subtype manifested as abnormal glycosaminoglycan metabolism, while the C4 subtype shows an obvious inflammatory response. In another study, Almeida et al. identified two OA subtypes (cluster A and cluster B) with unique pathophysiological processes and clinical phenotypes by integrating transcriptomic data with clinical data.^105^ Cluster A is enriched in cartilage-related pathways, while Cluster B shows marked upregulation of chemokine activity pathways. Cluster B also presents more severe joint space narrowing and lower osteophyte scores in the clinic, which were consistent with the molecular classification results. Similarly, Steinberg et al. conducted an RNA sequencing analysis of low-grade cartilage (showing no/minimal degeneration, cartilage normal/softening only) and synovial tissues from 113 OA patients.^106^ They identified two molecular subgroups: a highly inflamed subtype significantly linked to females and the use of proton pump inhibitors, and a low-grade inflamed subtype dominated by extracellular matrix/cell adhesion pathways. Xue et al. based on the gene expression profiles of different knee joint tissues, including cartilage, synovium, subchondral bone, and meniscus, and used unsupervised clustering analysis to classify OA into bone remodeling subtype, immune-metabolic subtype, and cartilage degradation endotypes.^107^ This classification takes into account the molecular heterogeneity and crosstalk of different tissues, and it attempts to correlate molecular characteristics with clinical phenotypes. For example, the bone remodeling endotype may be associated with changes in bone mass and osteophyte formation in OA patients; the immune-metabolic endotype may reflect the persistent immune-inflammatory response within the joint; the cartilage degradation endotype mainly involves pyroptosis and cell death processes, resulting in severe cartilage degradation. These molecular-based subtyping classifications reflect the heterogeneity of core driver pathways in the progression of OA and provide an important framework for understanding clinical variability among patients and developing targeted treatment strategies. They also suggest that the above-mentioned genetic heterogeneity may ultimately lead to different molecular subtypes by affecting these core molecular pathways.

#### Endotypes based on metabolomic profile

Metabolomic analysis is able to identify specific metabolic pathways and biomarkers related to OA, and thus provides a new perspective for OA classification.^108,109^ Carlson et al. used high-performance liquid chromatography-mass spectrometry (LC-MS) to conduct a metabolomic analysis of 75 synovial fluid samples from healthy individuals and OA patients at different stages. The results showed significant differences in the synovial fluid metabolome between healthy controls, early-stage OA, and late-stage OA. These differences were closely related to multiple metabolic pathways, such as structural damage, inflammation, oxidative stress, and vitamin metabolism. They also found that there were subgroups with different metabolic phenotypes within early- and late- stage OA patients.^110^ Hence, this study indicates that OA is a complex syndrome composed of multiple metabolic states. The targeted phospholipidomics study by Rocha et al. also supports this point. They found that the levels of multiple phospholipid species in the synovial fluid of OA patients were significantly elevated, and there were significant differences in the phospholipid profiles among different OA subgroups.^111^ Subsequently, Werdyani et al. also identified three endotypes of OA patients through plasma metabolomic analysis. These endotypes were defined by specific metabolite levels, including the myopathy-like type (high butyryl-carnitine C4 level), the arginine-deficient type (low arginine level), and the low-inflammatory type (low lysophosphatidylcholine level). There were associations between these three endotypes and the clinical as well as epidemiological characteristics of patients. For example, OA patients with the myopathy-like type often have higher BMI, prevalence of diabetes, and coronary heart disease, while OA patients with the arginine-deficient type are more likely to have coronary heart disease.^91^

## Heterogeneity in treatment and theratypes

OA management adheres to a stepwise principle, which involves a structured and progressive approach based on the severity of the disease and the individual needs of the patient. This approach typically begins with non-pharmacological interventions, such as patient education, lifestyle modifications, and physical therapy, to alleviate symptoms and improve joint function.^112,113^ In terms of pharmacological treatment, NSAIDs and acetaminophen are commonly used to alleviate symptoms. Additionally, intra-articular steroid injections can be employed as a short-term pain relief measure. For patients with advanced disease, total knee replacement surgery becomes a viable treatment option. The entire treatment process should be tailored to the specific circumstances of each patient.^112,114^ Although gender differences may lead to variations in the clinical presentation of OA, studies have shown that there is no prominent difference in treatment outcomes between genders after attempting various pharmacological treatments.^115^ However, the diversity of treatment responses remains a notable issue.^116^ Patient response to specific treatments is highly dependent on their predominant endotype/phenotype. For example, anti-inflammatory treatments (e.g., steroids and biologics) are efficient in patients with an inflammatory phenotype but may be ineffective in non-inflammatory phenotypes; metabolic interventions (e.g., weight loss and GLP-1 agonists) are crucial for obesity-related OA, while treatments targeting structural damage or pain pathways have their specific focus. Therefore, understanding the heterogeneity of treatment response is crucial for distinguishing OA theratypes and improving patient prognosis through targeted interventions.

Research into clinical phenotypes and molecular endotypes has significantly advanced the understanding of OA treatment subtypes, namely theratypes. These clinical or molecular markers discovered from phenotype and endotype enable the identification of patient subgroups that are more likely to benefit from or experience adverse effects with specific treatments, thereby facilitating intervention selection and standardizing treatment decision-making. Moreover, they are essential for developing targeted therapeutic strategies, advancing drug discovery, and improving prognostic predictions. For example, post-traumatic subtype OA can be targeted with protease inhibitors to inhibit pathological cascade reactions, metabolic subtype OA can be treated with anti-resorptive therapy to correct bone metabolic imbalance, and inflammatory subtype OA can be managed with anti-inflammatory therapy to block inflammatory signaling pathways.^117^ This, in turn, guides the selection of treatment candidates and the strategic stratification of treatment approaches.^114^

### Different subtypes and potential therapies

The following content will explore the heterogeneity of treatment responses in OA, focusing on inflammation-driven, obesity-related, metabolic, and structural damage subtypes. By aligning current therapeutic strategies with these specific subtypes, we aim to illuminate the nuanced differences in treatment efficacy and underscore the significance of personalized management for OA patients.

#### Inflammation-driven subtype: targeted biologics and anti-inflammatory therapy

NSAIDs are recommended as the first-line pharmacotherapy for OA pain management, particularly for knee and hip OA.^118^ The efficacy of different NSAIDs (e.g., COX-2 inhibitors, acetic acid derivatives, and propionic acid derivatives) in the treatment of OA is relatively small. However, the long-term use of NSAIDs is associated with a higher risk of gastrointestinal adverse reactions, and thus clinicians need to consider multiple factors, including the patient’s pain level, joint function, and gastrointestinal risk.^119^ Interestingly, treatment responses to NSAIDs are different among patients with varying joint involvement. A systematic review highlights moderate clinical heterogeneity in the efficacy of NSAIDs when comparing studies involving both hip and knee OA with those focusing exclusively on knee OA.^120^ Specifically, NSAIDs such as naproxen provided more substantial pain relief in knee OA compared to hip OA. Patients with knee OA exhibited improvements of 4 to 7 mm on the Western Ontario and McMaster Universities Osteoarthritis Index (WOMAC) pain score, stiffness, and function subscales, which were statistically significant relative to those with hip OA. The effect size for knee OA was approximately 0.8 (considered as a large effect), whereas the effect size for hip OA ranged from 0.5 to 0.6 (considered as a moderate effect). Additionally, both patients and clinicians consistently rated naproxen more favorably for the treatment of knee OA, suggesting the differential treatment effects across distinct joints.^121^ This significant joint-specific heterogeneity (knee > hip) may be closely related to differences in the distribution of OA subtypes. Knee OA is more commonly associated with synovial inflammatory features (e.g., synovial hyperplasia and inflammatory exudate), with pain that is sensitive to COX inhibition; in contrast, hip OA more frequently presents as a mechanical stress subtype, with pain potentially arising more from biomechanical changes and structural damage, and a relatively limited response to anti-inflammatory agents.^114,122^ In addition, the pain mechanisms in OA are complex and subtype-dependent. For example, in the inflammatory subtype, the synovial tissue is highly inflamed, producing large amounts of prostaglandin E2 (PGE2), and inhibiting COX enzymes (especially COX-2) can effectively alleviate pain.^123,124^ However, in mechanical stress subtype or metabolic subtype, joint pain may be more attributable to structural damage (e.g., osteophyte friction or subchondral bone changes) or neuropathic alterations, resulting in a relatively weaker response to NSAIDs.

Intra-articular corticosteroid injections (IACS) are widely used in the treatment of OA. Corticosteroids can bind to intracellular receptors, triggering a series of anti-inflammatory mechanisms that inhibit synovitis and joint effusion, thereby providing short-term relief of OA-related pain without inducing structural changes. Consequently, IACS may only be effective for pain relief and cannot improve joint function.^125^ This suggests that IACS may be only effective for inflammatory subtypes of OA (e.g., patients with obvious synovitis). Therefore, not all patients benefit from IACS. IACS are ineffective for thumb and finger joint OA.^126^ The proportion of pain relief from IACS varies, ranging from approximately 62% to 71%, with differences observed among patients in the degree and duration of relief. If a patient has subchondral insufficiency fractures, the injection may temporarily reduce pain and potentially increase the weight-bearing capacity, but it may exacerbate subchondral insufficiency fractures, leading to osteochondral defects, joint collapse, and intensified pain exceeding the pre-injection levels.^127^

Traditional anti-inflammatory drugs often fail to accurately target the joint microenvironment and thus cause undesired systemic adverse effects.^128^ Biologics can selectively modulate inflammation-related signaling pathways and responses, offering a promising therapeutic strategy for OA.^129^ Tumor necrosis factor (TNF) inhibitors have succeeded in patients with inflammatory knee OA^130^ and slowed the structural deterioration of inflammatory hip OA.^131^ However, efficacy is not obvious in patients without inflammation.^132^ Moreover, TNF inhibitors can slow the progression of hand OA in the distal interphalangeal joints, but they do not reduce the risk in the proximal interphalangeal joints or the occurrence of hand OA after 10 years.^133^ This heterogeneity stems from the differing pathological roles of TNF-α in various OA subtypes. In inflammatory subtypes, it acts as a key pro-inflammatory factor driving synovial hyperplasia and cartilage degradation; whereas in non-inflammatory or mechanical subtypes, cartilage degradation may be induced more by matrix metalloproteinases (MMPs) rather than TNF-α. Moreover, the lack of dynamic biomarkers limits its precise application. IL-1α and IL-1β are pro-inflammatory cytokines that share functional similarities with TNF-α in joint inflammation.^134^ Preclinical studies have shown that IL-1 pathway inhibitors can significantly ameliorate both symptoms and structural damage in animal models of OA.^135^ However, the translation of IL-1 inhibitors into clinical trials has yielded inconsistent results. Fleischmann et al found that the WOMAC score of knee OA patients at week 16 had improved significantly with lutikizumab 100 mg but not with the 25 mg or 200 mg doses compared to placebo, but there was no significant difference among all groups after 16 weeks. This suggests that there may be a dose-response threshold or inter-individual metabolic differences. Moreover, no significant differences were observed in the most structure-related endpoints and MRI-assessed synovitis.^136^ In another randomized controlled trial involving 170 patients with knee OA, participants were randomly assigned to receive either a single intra-articular injection of anakinra or a placebo. No statistically significant difference in WOMAC scores were observed between the anakinra and placebo groups at the 12-week follow-up.^137^ This may be related to uneven drug distribution or heterogeneity in IL-1 receptor expression among individuals with different subtypes of OA. The above findings collectively emphasize the necessity of implementing stratified treatment based on disease subtypes (e.g., the presence or absence of an IL-1-driven inflammatory phenotype), which provides key evidence for the optimization of precision anti-IL-1 treatment strategies.

DMOADs are regarded as the most promising approach for the future treatment of OA. The traditional DMARDs methotrexate (MTX) has been considered as a potential DMOAD because MTX shows efficacy in patients with MRI-confirmed synovitis.^138^ In the METHODS trial, MTX was proven to effectively alleviate the pain of hand OA.^139^ In another randomized, placebo-controlled clinical trial, oral administration of MTX was found to reduce pain and stiffness, and improve function at 6 months in knee OA patients.^140^ Nevertheless, our recent MESKO trial found that low-dose MTX was not effective in reducing effusion-synovitis or other symptoms in patients with mid- to late-stage knee OA.^141^ The differences in MTX efficacy suggest that it may be more effective for specific inflammatory subtypes (hand OA or knee OA with significant synovitis) and less effective for patients with non-inflammatory-dominant or late-stage structural damage, emphasizing the importance of patient selection based on OA subtype.

#### Obesity and metabolic subtypes: weight management and metabolic interventions

Exercise is a primary treatment of OA. Exercise alleviates osteoarthritis symptoms in a variety of ways, including strengthening the muscles around the joints, enhancing psychological resilience, reducing metabolic inflammation, and raising the pain threshold.^142^ Different types of exercise therapies show varying effects in relieving pain, stiffness, improving joint function, and enhancing quality of life. For example, aquatic exercises are efficient in relieving pain, while yoga is optimal in improving joint stiffness, functional limitations, and quality of life.^143^ Walking is one of the most common exercises and offers numerous health benefits. Lo et al. found that walking was associated with less frequent development of knee pain in OA patients who were age 50 years or older.^144^ However, Doré et al found that participants doing ≥10 000 steps/day had a greater risk of bone marrow lesions, increasing meniscal pathology score, and cartilage defect score.^145^ Recently, He et al. showed that daily purposeful walking steps (cadence ≥60 steps/minute) with a potential threshold near 8 000 steps/day were associated with lower incident symptomatic knee OA, whereas more daily unintentional walking steps (cadence <60 steps/minutes) were significantly associated with higher incident symptomatic knee OA.^146^ These studies suggest that the effect of exercise therapy may be influenced by various factors, including the type of exercise, intensity, frequency, and individual differences among patients. Based on Osteoarthritis Research Society International (OARSI) clinical practice guidelines, within a personalized care framework, patients with knee, hip, or multi-joint OA should prioritize structured land-based exercises (e.g., strength training, aerobic exercise, and tai chi), which can be used alone or in combination with other therapies; water-based exercises may be conditionally recommended for those without gastrointestinal/cardiovascular complications; for high-risk fall populations (e.g., frail individuals or those with balance disorders), the regimen should be adjusted to include seated exercises, activities assisted by walkers, or mind-body modalities like tai chi or yoga.^114^ This recommendation reflects the guiding value of treatment heterogeneity for precise interventions.

Obesity is a major risk factor for OA, and weight loss shows benefit in alleviating symptoms of knee OA. Glucagon-like peptide-1 (GLP-1) receptor agonists have shown promise in weight loss and are expected to serve as a weight loss-mediated therapy for knee OA, particularly suitable for patients with both type 2 diabetes mellitus and obesity.^147^ GLP-1 receptor agonists can not only achieve weight loss through central appetite suppression and delayed gastric emptying, but also have direct anti-inflammatory and possible chondroprotective effects,^148,149^ which may give them additional therapeutic value beyond simple weight loss. Very recently, a clinical trial assessed the efficacy of once-weekly subcutaneous injections of semaglutide (a typical GLP-1 receptor agonist) in obese OA patients with moderate to severe knee pain.^150^ The results demonstrate that semaglutide significantly reduced body weight and pain related to knee OA compared to placebo. However, the tolerability of weight loss medications, such as gastrointestinal adverse reactions, cannot be ignored, as they may affect treatment responses. Studies have shown that GLP-1 receptor agonists are associated with an increased risk of pancreatitis, intestinal obstruction, and delayed gastric emptying, and these gastrointestinal adverse reactions may reduce patient adherence to treatment and overall treatment effectiveness.^151^ Weight loss is also associated with symptom relief of hip OA in overweight or obese patients.^152^ It primarily exerts its protective effect by directly reducing the mechanical load on joints (especially weight-bearing joints) and slowing the rate of cartilage wear. This treatment method may be more suitable for patients with the metabolic subtype and the mechanical subtype. Weight loss has associated beneficial effects on hip OA even among participants aged 65 or older, but it should be noted that weight loss is associated with increased health risks in the elderly, such as an increased risk of hip fractures, functional impairment, accidental disability, and mortality.^153^ In addition, elderly patients often suffer from sarcopenia and decreased bone density. Therefore, weight loss strategies for this population must be cautious. It is essential to emphasize resistance training to maintain muscle mass and strength and to closely monitor bone density to avoid offsetting the benefits of weight loss. These age-related risk differences necessitate highly individualized treatment plans tailored to the patient’s specific subtype and overall condition.

Metformin is a first-line medication for type 2 diabetes and shows potential in OA treatment. The underlying mechanisms involve reducing chondrocyte apoptosis and degradation, as well as inhibiting the infiltration of synovial macrophages, thereby interfering with the progression of OA.^154,155^ For OA patients with diabetes, the use of metformin, as compared to other medications, can reduce the risk of developing OA.^156^ However, metformin is also another example of drug efficacy that is highly dependent on the patient’s metabolic status. Its potential benefits may be primarily limited to OA subtypes associated with metabolic dysfunction, while it may be ineffective for other subtypes.

#### Structural damage type: structural repair and disease-modifying therapy

Intra-articular hyaluronic acid injections (IAHA) have garnered increasing attention in recent years. Hyaluronic acid may exert chondroprotective effects through mechanisms such as restoring the concentration of hyaluronic acid in the joint, alleviating pain, improving function, delaying progression, and promoting chondrocyte proliferation and extracellular matrix synthesis.^157,158^ However, the efficacy of IAHA in OA remains controversial. Meta-analysis revealed that hyaluronic acid has a small effect when compared with an intra-articular placebo, but the highest-molecular-weight hyaluronic acid may be more efficacious in treating knee OA compared with lower-molecular-weight hyaluronic acid.^159^ A study involving 253 patients showed that hyaluronic acid derivative Hylan G-F 20 significantly improved the WOMAC pain score of patients over 26 weeks compared to placebo.^160^ In another Grade I non-inferiority trial (involving 321 patients, with 7 lost to follow-up), both high molecular weight hyaluronan produced by biological fermentation (Bio-HA) and avian-derived cross-linking hyaluronan (CL-HA) improved the pain and function scores of OA patients.^161^ However, IAHA does not improve the PROMIS scores of patients with knee OA.^162^ Another multicenter, randomized, placebo-controlled trial showed that a single IA injection of HA is no more effective than a placebo in treating the symptoms of hip OA.^163^ A high dose of IAHA may show better results than a low dose in some studies.^164^ Thus, heterogeneity of these results limits definitive conclusions of IAHA in OA therapy.

Platelet-rich plasma (PRP), a concentrate of platelets derived from a patient’s blood, contains various growth factors and cytokines that have shown potential in promoting cartilage repair and alleviating symptoms of OA. However, the effects of PRP therapy are closely related to the types of cytokines it contains. Cytokines such as TGF-α and β, PDGF, EGF, and IGF-1 have potential protective effects on OA, while cytokines like vascular endothelial growth factor (VEGF) and TNF-α may induce negative effects on OA. Thus, it could be one of the main reasons for the heterogeneity observed in PRP therapy.^165^ Bennell et al. showed that intra-articular injection of PRP did not result in a significant difference in symptoms or joint structure of patients with symptomatic mild to moderate radiographic knee OA at 12 months as compared with saline placebo.^166^ In another trial, although PRP injections are more effective than IAHA in the short term, no obvious differences have been observed in the long term.^167,168^ In addition, PRP is combined with other treatments. PRP injections alone demonstrated a superior efficacy in improving joint function compared to PRP combined with IAHA, IACS, IAHA alone, or placebo. Conversely, in terms of pain relief, the combination of PRP and IAHA is shown to be more effective than other single treatments or placebo.^159^

Stem cell therapy has drawn growing attention in OA treatment because stem cells are multipotent progenitor cells with self-renewal and multi-lineage differentiation properties, and can be derived from bone marrow, peripheral adipose tissue, blood, etc.^169,170^ It has been demonstrated that stem cell therapy is safe for OA treatment with mild complications such as joint swelling and injection site pain.^171^ Intra-articular injection of allogeneic adipose-derived mesenchymal cells is shown to improve pain and knee function in knee OA patients, but there are differences in treatment doses and effects among different studies. The source of stem cells may also affect the therapeutic effect. In the study by Chen et al., 11 subjects received intra-articular injections of high-dose (4 × 10^7) and low-dose (6.7 × 10^6) adipose-derived mesenchymal stem cells. The results showed that both the low-dose and high-dose groups exhibited a trend of improvement in pain and knee function after treatment.^172^ However, another study has found that injection of bone marrow-derived mesenchymal stem cells (1 × 10^7) can lead to the formation of scar tissue-free bodies, resulting in joint dysfunction.^173^ This heterogeneity may be due to stem cell manipulation and sources. For example, adipose mesenchymal stem cells derived from obese patients may have lower proliferative capacity and increased expression of senescence and pro-inflammatory cytokines, which may affect treatment outcomes.^174^ Therefore, further research is needed to investigate the optimal dose and source of stem cells. For individualized treatment design, it is necessary to combine biomarker classification with optimization of stem cell sources and doses for different pathological subtypes of OA.

Sprifermin, recombinant human fibroblast growth factor 18 (rhFGF18), represents another promising DMOAD. The FORWARD trial demonstrated that sprifermin not only significantly increased cartilage thickness but also effectively alleviated pain symptoms in knee OA, with sustained efficacy observed for up to 5 years.^138^ This suggests its potential long-term therapeutic effect in OA management. Further studies showed that sprifermin enhances cartilage thickness in the femoral-tibial joint, particularly in high-load-bearing regions such as the central medial tibia.^175^ Interestingly, basic research indicates that the chondrogenic effects of sprifermin in cartilage explants ex vivo are partially dependent on the inflammatory microenvironment.^176^ In addition, Valsesia et al. have performed a retrospective analysis of sprifermin clinical trial data and found that two combinations of SNPs in the interleukin 1 receptor antagonist (IL-1RN) gene may be associated with disease severity, progression, and the potential response of specific genetic populations to sprifermin.^177,178^ They have identified four groups of patients and tested the statistical association between these groups and changes in cartilage thickness and volume (measured by MRI) and WOMAC scores. Patients with the SNPs rs9005 and rs315952 show a better response to sprifermin treatment in terms of cartilage volume.^177^ Hence, the therapeutic efficacy of sprifermin appears to vary across different patient subtypes,^179^ and it may be more effective in OA patients with mild synovitis or low levels of pro-inflammatory factors, as well as specific genetic backgrounds, but further clinical research is needed.

### Theratypes of OA

It should be noted that OA theratypes are not static. As the disease progresses, patients’ clinical phenotypes and molecular endotypes may evolve, necessitating repeated reassessment and adjustment of treatment strategies.^92^ For example, in post-traumatic OA, patients may initially present with a predominantly mechanical or structural phenotype characterized by joint instability, focal cartilage injury, or altered loading patterns.^180,181^ However, persistent tissue damage can activate the synovium and lead to the release of inflammatory mediators, resulting in a shift toward a more inflammation-dominant state over time.^182^ Similarly, patients with metabolic OA may initially present with obesity, metabolic syndrome, and low-grade systemic inflammation, but later experience more pronounced synovitis and exacerbated pain.^183,184^ In certain instances, patients with relatively minor structural damage may progressively develop disproportionate pain, widespread pain distribution, or characteristics of central sensitization, indicating a shift toward a pain-processing-dominant theratype.^185,186^

Consequently, the establishment of a dynamic and flexible treatment system is critical for achieving precision medicine in OA management. However, research on OA theratypes remains limited, and a comprehensive understanding has yet to be established. At present, several candidate biomarkers and assessment modalities have been associated with major OA subtypes, including inflammatory cytokines and synovitis detected by imaging for inflammation-related OA, metabolic parameters and adipokines for metabolic OA, cartilage degradation markers such as C-telopeptide fragments of type II collagen (CTX-II) or collagenase generated carboxy-terminal neoepitope of type II collagen (C2C) for tissue catabolism, and quantitative sensory testing or widespread pain patterns for pain-sensitization states.^184,187–190^ However, most of these markers remain exploratory or associative, and few have been prospectively validated as predictive biomarkers for guiding treatment in routine clinical practice.^190,191^ Consequently, while the identification of clinical phenotypes and molecular endotypes offers potential for the development of OA theratypes, the existing evidence is insufficient to support a universally accepted theratype classification system for clinical decision-making.^191^

This gap is likely attributable to the complexity of OA’s underlying mechanisms, the significant variability in individual treatment responses, and methodological limitations in current research. Specifically, genetic factors play a pivotal role in the onset of OA, but the contribution of individual gene variants to disease risk is relatively modest, and the interactions among multiple genes further complicate the genetic landscape.^5^ Environmental factors, including age, occupation, and lifestyle habits, are also closely associated with OA onset.^126,192–194^ The interactions among these environmental factors and genetic factors add layers of complexity to the pathological processes and treatment responses in OA. Furthermore, the substantial variability among individuals leads to significantly different responses to the same interventions. These overall factors make it challenging to categorize patients into distinct theratypes using a simplistic classification method and a unified standard. In sum, while the identification of clinical phenotypes and molecular endotypes holds promise for the development of OA theratypes, the field faces significant challenges due to the disease’s multifactorial nature and the heterogeneity of patient responses.

## Challenges and limitations of the current classification system

**Fig. 2:**
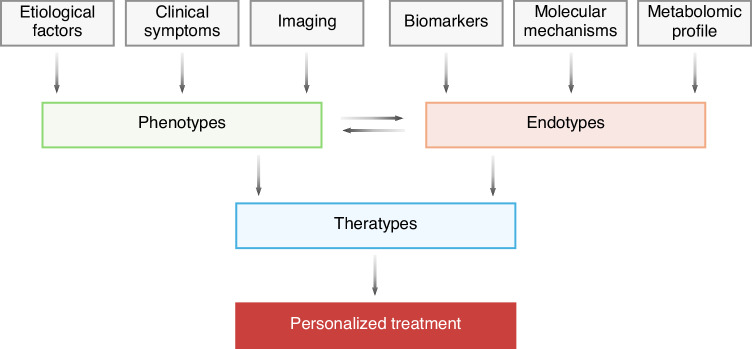
Classification of OA. The phenotype classification of OA is based on a range of clinical factors, including etiological determinants, clinical manifestations, and imaging characteristics. Endotypes are discovered through the analysis of biomarkers, molecular pathways, and metabolomic profiles of blood and joint tissues. The interrelationship between phenotypes and endotypes provides the foundation for defining OA theratypes, paving the way for personalized treatment strategies

So far, the standards of OA classification are not uniform. Based on a review and analysis of existing literature, we summarized current research on OA classification (Fig. 2 and Table 1), aiming to provide a scientific and standardized reference framework. Despite significant advances in phenotypic and endotypic OA, the classification of OA still faces enormous challenges and limitations. First, the molecular and clinical phenotypes of OA are diverse and interrelated. For example, inflammatory OA patients present with persistent and progressive pain, pathologically driven by synovial macrophage infiltration and elevated inflammatory cytokine expression, involving inflammatory responses and joint tissue degradation, with MRI showing synovial thickening and effusion-synovitis.^49,50,90,96^ The current core challenge lies in accurately identifying clinical phenotypes and clarifying their molecular endotypes. In-depth analysis of underlying molecular mechanisms will drive the development of precision medicine tools for predicting disease progression, guiding treatment classification, and optimizing intervention strategies. However, the complexity of OA mechanisms makes it difficult for existing classification systems to fully capture the essence of the disease, hindering the translation of these classifications into powerful clinical tools and universally applicable frameworks.

**Table 1:** Current research on OA classification

Classification hierarchy	Classification basis	Reference	Subtype category	Key characteristics	Classification methods	Analyzing from clinical samples	Statistical information
Phenotypes	Etiological Factors	Herrero-Beaumont et al.^35^	1. Genetically determined 2. Estrogen-dependent 3. Aging-related	Emphasizing genetic mutations, estrogen levels, and aging process.	A review of the literature was performed by searching the Medline and PubMed databases from 1952 to November 2008.	No	n/a
		Mobasheri et al.^36^	1. Synovitis-driven inflammatory 2. Metabolic 3. Age-driven 4. Cartilage-driven 5. Trauma-induced injury-driven 6. Subchondral bone-driven	Considering from the perspective of disease-driven factors.	Summarizing phenotypes from previous studies.	No	n/a
		Huang, C. et al.^38^	1. Load-bearing 2. Structural 3. Inflammatory 4. Metabolic 5. Systemic factor 6. Mixed	A comprehensive framework for clinical and research. Each type has a specific etiology, pathogenesis, and treatment strategy.	This study conducted questionnaire survey and literature review. COACH Panel members were invited to review the drafted classification criteria and optimize classification criteria.	No	n/a
	Clinical Symptoms	Kittelson, A. J. et al.^49^	1. Numerous comorbidities 2. High knee joint sensitivity 3. Psychological stress and severe pain 4. Mild radiological manifestations, good mental health, strong muscle strength, low pain sensitivity, the youngest age, and the least functional impairment	Considering OA-related indicators, including pathology, psychological distress, and altered pain neurophysiology.	Latent Class Analysis was applied to the following variables: radiographic OA severity, quadriceps strength, BMI, Charlson Comorbidity Index (CCI), Center for Epidemiologic Studies Depression subscale (CES-D), Coping Strategies Questionnaire-Catastrophizing subscale (CSQ-Cat), number of bodily pain sites, and knee joint tenderness at 4 sites.	Yes	Data were obtained from a total of 3 494 participants at Visit #6 of the Osteoarthritis Initiative (OAI) study. Class 1 constituted 4% of the study population, Class 2 comprised 24%, Class 3 represented 10%, and Class 4 accounted for 62%.
		Vongsirinavarat. et al.^55^	1. No disability 2. Mild disability 3. Moderate disability 4. Severe disability	There are significant differences among various phenotypes in parameters such as pain intensity, range of motion, muscle power, and participation restriction levels.	Cluster analysis was utilized to identify variables associated with activity limitations. Physical examinations were performed on patients with symptomatic knee OA. Eligible OA patients were then classified according to the clustering variables.	Yes	A total of 250 participants with symptomatic knee OA were enrolled in this study. The results identified four phenotypes related to the extent of activity limitation: no disability (31.6%), mild disability (26.8%), moderate disability (30.4%), and severe disability (11.2%).
		Perrot, S. et al.^56^	1. Local pain 2. Neuropathic-like pain 3. Deep pain	Evaluating various symptoms of OA pain through qualitative descriptive terms can effectively differentiate and quantify the three clinically relevant dimensions of OA pain.	The Osteoarthritis Symptom Inventory Scale (OASIS) was developed and validated to assess various dimensions of OA pain symptoms related to the mechanisms of OA pain, thereby facilitating the qualitative classification of OA.	Yes	This study enrolled 123 patients from September 2016 to March 2018. Among these, 118 patients were included in the study, while 5 patients were excluded.
	Imaging Features	Roemer, F. W. et al.^50^	1. Inflammatory 2. Meniscus-cartilage 3. Subchondral bone 4. Atrophic 5. Hypertrophic	Standardized MRI assessment framework. Inflammatory and subchondral phenotypes are significantly associated with progression and TKR.	The Rapid OsteoArthritis MRI Eligibility Score (ROAMES) was utilized to screen and stratify potential candidates for knee joint assessment into distinct structural phenotypes while documenting the relevant diagnoses that lead to exclusion.	Yes	ROAMES identified 43 of 50 [86%, 95% CI: (73%, 94%)] pre-defined phenotypes correctly. 10 of 10 (100%) into cluster 1, 10 of 10 (100%) into cluster 2, 9 of 10 (90%) into cluster 3, 6 of 10 (60%) into cluster 4 and 8 of 10 (80%) into cluster 5.
		Park, S. Y. et al.^72^	1. Unicompartmental 2. Tricompartmental (a) Tricompartmental joint space narrowing-dominant (b) Tricompartmental osteophyte-dominant	AI models identify microheterogeneity and complex patterns, enabling high-precision prediction of OA onset and progression.	This study employed machine learning to identify key factors associated with various patterns of OA progression. Data from the clinical data warehouse (CDW) of a single institution were collected from 79 634 patients who visited the outpatient clinic for knee pain between April 2003 and October 2017. Patients were classified based on imaging data obtained from the final follow-up X-rays.	Yes	A total of 833 knee data points were finally included and utilized to develop a model for predicting the progression of knee OA: Cluster 1 (*n* = 479) and Cluster 2 (*n* = 354). However, this model has yet to be validated in external cohorts.
Endotypes	Biomarkers	Mobasheri, A. et al.^86^	1. Inflammatory	1. IL-1β, IL-6, TNF-α ↑	This study conducted a literature review to explore the molecular phenotypes of OA based on biomarkers.	No	n/a
			2. Subchondral bone remodeling	2. CTX-I (bone resorption) ↑			
			3. Metabolic syndrome	3. Abnormal metabolic markers			
			4. Aging-related	4. ECM changes, pentosidine accumulation			
		Lv, Z. et al.^92^	1. Early-stage pain 2. Structural lesion with pain (a) Cartilage degradation-driven (b) Bone remodeling-driven (c) Inflammation-driven (d) Pain-driven 3. Late-stage pain	Unique patterns of change in WOMAC pain, JSW, cartilage thickness, and osteophytes over time. The subtypes derived from structural lesions with pain are characterized as followed: (a) Imbalance of cartilage matrix; (b) Changes in bone remodeling markers; (c) Inflammatory markers ↑; (d) Linked to BMLs/synovitis.	This study conducted a literature review based on the temporal alterations of representative molecules and the major pathophysiology in patient clusters to classify patients.	No	n/a
		Angelini, F. et al.^96^	1. Low tissue metabolism (C1)	1. Lower levels of inflammation/structural markers	The IMI-APPROACH cohort screened 433 patients with OA and enrolled 297 individuals who were most likely to experience pain and/or structural progression at the 2-year follow-up. Classification models were trained to predict cluster membership, and Explainable AI techniques were used to interpret these to reveal the driving factors behind each cluster. These methods were applied to the IMI-APPROACH cohort to identify distinct phenotypes.	Yes	Among the enrolled patients, the majority were women (*n* = 230) and predominantly Caucasian/white (*n* = 283). This model has been validated in external cohorts (FNIH/OAI).
			2. Structural damage (C2)	2. Bone/cartilage degradation markers ↑			
			3. Systemic inflammation (C3)	3. Inflammatory response ↑ and tissue degeneration ↑			
	Molecular Mechanisms	Dell’Isola, A. et al.^99,100^	1. Chronic pain	1. Central sensitization	A systematic literature search was conducted in PubMed (Medline), resulting in the inclusion of 26 studies for systematic evaluation and classification. Verification was performed using the OAI database.	Yes	599 patients were selected from the OAI database FNIH at 24 months’ time to conduct the study. Phenotype allocation was successful for 84% of cases with an overlap of 20%.
			2. Inflammatory	2. Inflammatory biomarkers ↑			
			3. Metabolic syndrome	3. Linked to obesity/diabetes			
			4. Bone and cartilage metabolism abnormality	4. Local metabolic changes			
			5. Mechanical overload	5. Joint mechanical abnormalities			
			6. Minimal joint disease	6. Mild symptoms			
		Han, Z. et al.^39^	1. Inflammation-dominant	1. High levels of inflammatory cytokines and synovitis	This study focused on the mechanisms and hallmarks of cellular senescence through a literature review, summarizing evidence that supported the relationship between cellular senescence and inflammation.	No	n/a
			2. Cellular senescence-dominant	2. Chondrocyte senescence			
		Yuan, C.^104^	1. Glycosaminoglycan metabolism disorder (C1)	1. Abnormal glycosaminoglycan metabolism;	This study collected three types of tissue (cartilage, subchondral bone, and synovium) from multiple clinical centers to construct a comprehensive transcriptome atlas of OA patients. Through unsupervised clustering analysis of the cartilage transcriptome, OA patients were categorized into four subtypes.	Yes	131 OA patients were classified into four subtypes: 81 (61.8%) into cluster 1 (C1), 24 (18.3%) into cluster 2 (C2), 10 (7.6%) into cluster 3 (C3) and 16 (12.2%) into cluster 4 (C4).
			2. Collagen metabolism disorder (C2)	2. Abnormal collagen metabolism			
			3. Activated sensory neuron (C3)	3. Sensory neuron activation			
			4. Inflammatory (C4)	4. Obvious inflammatory response			
		Xue, Y.^107^	1. Bone remodeling	1. Associated with bone mass changes	This study obtained gene expression profiles of cartilage, synovium, subchondral bone, and meniscus from the Gene Expression Omnibus (GEO) and employed unsupervised clustering to categorize OA into three distinct subtypes.	Yes	A total of five microarray raw datasets were utilized, comprising 20 cartilage samples from OA patients, 20 synovial samples from the same platform, 40 subchondral bone samples, and 12 meniscus samples. The 92 OA tissue samples were categorized into three subtypes: Cluster 1 (*n* = 29), Cluster 2 (*n* = 32), and Cluster 3 (*n* = 31).
			2. Immune-metabolic	2. Persistent immune-inflammatory response			
			3. Cartilage degradation	3. Pyroptosis, severe cartilage degradation			
	Metabolomic Profile	Werdyani, S.^91^	1. Myopathy-like	1. High butyryl-carnitine C4 level	This study conducted a metabolomic analysis of fasting plasma samples from patients and employed common factor analysis and K-means clustering to identify phenotypes of OA patients based on the metabolomic data.	Yes	A total of 615 primary OA patients and 237 controls were included in the study. The 615 OA patients were categorized into three clusters: Cluster 1 (66 patients), Cluster 2 (200 patients), and Cluster 3 (349 patients).
			2. Arginine-deficient	2. Low arginine level			
			3. Low-inflammatory	3. Low lysophosphatidylcholine level			
Theratypes	n/a	n/a	Current research on OA theratypes remains scarce	A treatment subtype based on clinical phenotypes and molecular endotypes, aiming to match the right patient with the right therapy	n/a	n/a	n/a

*n/a* not applicable

Second, current OA classifications are characterized by significant heterogeneity in methods and standards. Phenotypic classifications, while valuable, typically rely on single dimensions such as etiology,^35^ clinical symptoms,^49^ or imaging features.^50^ Similarly, endotype classifications primarily focus on isolated molecular pathways like inflammatory biomarkers,^90^ metabolic features,^91^ or transcriptomic profiles.^104^ Unidimensional classification approaches often overlook the intrinsic interconnections among OA dimensions, leading to conflicting and overlapping categorizations across studies. For instance, the COACH framework emphasizes multifactorial mixed phenotypes, contrasting with simpler etiological models like Herrero Beaumont’s.^35,38^ The low-repair phenotype may represent an overarching category encompassing age-related phenotypes, potentially overlapping with diverse subtypes and correlating with rapidly progressive OA or requiring multi-mechanism targeted therapies. Similarly, mechanical phenotypes can trigger multiple molecular pathways; for example, cartilage phenotypes may transition to inflammatory states, particularly in obesity-related phenotypes. Systemic inflammatory endotypes identified by Angelini et al. and metabolomics-defined hypo-inflammatory endotypes from Werdyani et al. show distinct associations with inflammatory biomarkers and palmitoyl phosphatidylcholine, with partial overlap with inflammatory phenotypes in the COACH classification.^91,96^ In addition, emerging theratype-based frameworks should also be interpreted cautiously, as they remain largely conceptual at present. Although theratypes are intended to support treatment matching according to dominant disease-driving mechanisms, these categories are unlikely to remain stable throughout the disease course.^191,195^ Patients may shift from one dominant phenotype or endotype to another during disease progression, yet evidence of such theratype switching in well-characterized OA cohorts remains limited.^195–197^ Thus, the lack of real-world validation reduces the stability, interpretability, and clinical applicability of current classification systems.^195^

Third, current research on biomarkers, molecules, and metabolism related to endogenous classification of OA is mainly based on cross-sectional data, while longitudinal analysis of their dynamic changes has not yet been fully conducted.^90^ Dynamic monitoring of biomarkers can provide real-time assessment of drug efficacy or early validation of compound efficacy in interventional trials.^198–200^ Longitudinal monitoring of individual biomarkers may have limited predictive power for the progression of OA, but they can serve as patient stratification tools and have certain uses in enriching progressive OA trials.^96,201^ Future research could further explore longitudinal data on biomarkers, imaging tests, and other indicators of OA to further refine the description of phenotypes and possibly explore more detailed stratification methods. Although multiple candidate biomarkers have been associated with specific OA subtypes, very few have been prospectively validated as predictive biomarkers for treatment choices.^202^ Namely, the existing evidence is inadequate to establish a reliable connection between a given subtype and a corresponding intervention strategy, or to predict preferential therapeutic responses in routine clinical practice. Thus, the current classification framework, including theratype-oriented stratification, still has limited capacity to support precise treatment matching and personalized intervention.^191,195^

Finally, the complexity and diversity of existing classification methods, coupled with the absence of a unified and user-friendly framework, may hinder their application and acceptance rate in clinical practice.^38,203^ The adoption of certain advanced classification-related diagnostic techniques and treatments will definitely incur substantial costs.^204^ For instance, utilizing high-end imaging equipment for precise phenotype identification and developing targeted therapies based on specific endotypes can be financially burdensome.^196,204^ These financial issues will also restrict the widespread use and promotion of current OA classification systems, particularly in resource-limited areas.^196,204,205^

## Perspectives of precision treatment for OA

With the advancements in artificial intelligence and big data technologies, investigators and clinicians can integrate multi-omics approaches and clinical data, paving the way for significant breakthroughs in exploring the molecular mechanisms and identifying novel therapeutic targets. Currently, there is a notable deficiency in the availability of efficient genetic or multi-omics tools capable of stratifying OA patients for personalized treatment. This required the amalgamation of multidimensional data, including patients’ genetic profiles, lifestyles, clinical manifestations, imaging data, and biomarkers, coupled with the application of ML algorithms to develop predictive models.^206^ Such a data-driven approach will facilitate the precise identification of patient subgroups that exhibit favorable responses to specific therapeutic interventions.

Investigation into OA heterogeneity and classification are expected to promote future drug development with a focus on targeted therapies and multi-target strategies. The integration of pharmacogenomics will facilitate personalized medications for individuals and enable real-time monitoring of therapeutic responses, thereby optimizing treatment efficacy.^207,208^ In addition, given the gap between existing research models and the natural progression of OA, investigators and clinicians must judiciously select models appropriate for preclinical studies and conduct precise clinical trials on specific OA subtypes. The US Food and Drug Administration (FDA) recently announced plans to phase out animal research in drug testing, making animal models no longer mandatory for preclinical drug testing. Clinical trials for investigational therapies based on preclinical efficacy data from organ-on-a-chip (OoC) results combined with existing safety data, without animal testing, are undergoing.^209^ The establishment of a body-on-a-chip system can facilitate studies on how other organ systems, such as the gut microbiome and the central/peripheral system, affect OA development and drug responses.^210^ Future breakthroughs in personalized BoCs will further unleash the great potential of OoCs in drug development, paving the way for new strategies for precise OA treatment.

**Fig. 3:**
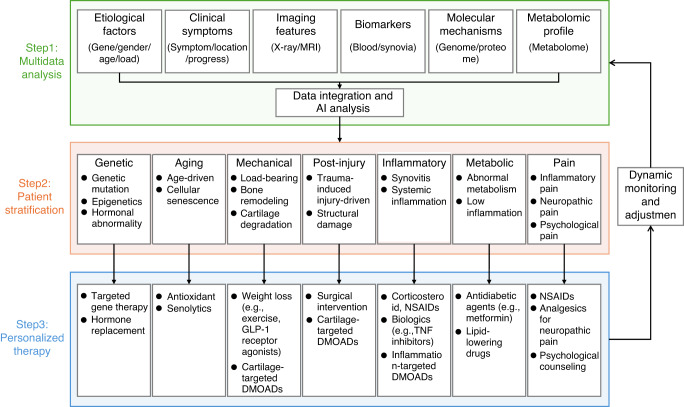
Conceptual framework for personalized intervention of OA. This framework facilitates precise intervention for OA patients through three stages: multidata analysis, patient stratification, and personalized therapy. By integrating clinical phenotypes, molecular endotypes, and theratypes across multiple dimensions, this framework shows the potential for addressing the limitations of traditional treatment strategies. Through dynamic monitoring and treatment adjustment, personalized treatment can be further promoted. It should be noted that this framework only presents some typical examples. Some patients may have mixed subtypes and may benefit from a combination of treatments

The advances in OA heterogeneity and classification will revolutionize the traditional “one-size-fits-all” treatment paradigm. Accurately stratifying patients will facilitate the transition from an experience-driven approach to a personalized intervention mode.^191,211^ This transformation is crucial for the development of effective DMOADs. The failures observed in previous DMOAD clinical trials are frequently linked to patient selection bias,^212^ and precise stratification can mitigate this issue. Specifically, by integrating clinical assessment data, imaging features, biomarker indicators, multi-omics techniques, and AI techniques, dynamic stratified models will be developed in the future.^213–217^ These systems will aim to identify the clinical phenotypes of OA alongside molecular endotypes, and ultimately determine theratypes, enabling accurate predictions of patient responses to specific interventions (Fig. 3). This personalized treatment approach is anticipated to significantly improve the therapeutic efficacy for OA, minimize side effects associated with unnecessary medications, and effectively reduce the overall disease burden.

## Conclusions

Advances in both clinical and basic research have revealed that OA exhibits high heterogeneity with a variety of genetic factors, pathophysiological processes, clinical manifestations, treatment responses, and prognostic features in different patients, joints, and stages of the disease. A deeper understanding of OA heterogeneity helps to identify characteristic changes and evolutionary trajectories of different subtypes. The classification of OA has evolved from the conventional radiological finding-based system to a multi-dimensional strategy that integrates etiological factors, clinical symptoms, imaging changes, molecular mechanisms, and biomarkers. These classification methods facilitate early diagnosis of the disease and provide a scientific basis for personalized treatment. However, significant challenges remain in OA classification. Existing classification methods lack a unified standard and need validated detectable biomarkers. Additionally, phenotypes and endotypes often overlap. Findings from subtype research have been difficult to apply in clinical practice. By fully understanding the heterogeneous characteristics and establishing optimized classifications of OA, the development of precise and personalized therapies for OA patients can be accelerated.
